# Barriers in providing primary care for immigrant patients with dementia: GPs’ perspectives

**DOI:** 10.3399/bjgpopen18X101610

**Published:** 2018-10-03

**Authors:** Rosa Vissenberg, Ozgul Uysal, Miriam Goudsmit, Jos van Campen, Bianca Buurman-van Es

**Affiliations:** 1 GP and Postdoctoral Researcher, Department of Internal Medicine, Academic Medical Centre, Section of Geriatric Medicine, Amsterdam, The Netherlands; 2 Neuropsychologist and Postdoctoral Researcher, Department of Internal Medicine, Academic Medical Centre, Section of Geriatric Medicine, Amsterdam, The Netherlands; 3 Clinical Psychologist, Department of Medical Psychology and Hospital Psychiatry, Medical Centre Slotervaart, Amsterdam, The Netherlands; 4 Geriatrician, Department of Geriatric Medicine, Medical Centre Slotervaart, Amsterdam, The Netherlands; 5 Registered Nurse and Professor, Department of Internal Medicine, Academic Medical Centre, Section of Geriatric Medicine, Amsterdam, The Netherlands

**Keywords:** dementia, cognitive dysfunction, general practice, emigrants and immigrants, culturally competent care, qualitative research

## Abstract

**Background:**

Dementia rates are growing rapidly in all regions of the world. In the Netherlands, the incidence of dementia among older immigrants will increase twice as fast compared with the native older population. It, therefore, needs special attention.

**Aim:**

To describe the barriers for providing primary care to immigrant patients (Turkish, Moroccan and Surinamese) with dementia from the perspectives of GPs.

**Design & setting:**

A mixed-method study, consisting of an online survey and focus groups.

**Method:**

An online survey was performed among 76 GPs working in the four biggest cities of the Netherlands. The barriers to providing primary care for immigrants with dementia were identified. Subsequently, three focus groups were carried out among 17 primary care physicians to discuss this topic further, and identify possible solutions and recommendations to improve dementia care.

**Results:**

GPs experience many obstacles in the care for the immigrant patient with dementia, namely in the diagnostic process, early detection, and assessment of care needs. Strong collaboration between primary care, community care organisations, specialised memory clinics, and municipalities is needed to optimise healthcare information provision, the availability of culturally sensitive facilities, and the enhancement of healthcare professionals' training and education.

**Conclusion:**

Important barriers were identified and recommendations were formulated for future healthcare policy. To be prepared and guarantee optimal care for the rising number of immigrant patients with dementia, recommendations should be implemented and effectiveness should be evaluated as soon as possible.

## How this fits in

For non-Western immigrant patients with dementia, research has primarily focused on prevalence, diagnostic screening, and caregiving. This is the first study to describe GP perspectives on providing care to this group. Important barriers were identified and recommendations were formulated for future healthcare policy.

## Introduction

Dementia rates are growing rapidly in all regions of the world owing to an ageing population and dementia is becoming a major public health priority. In 2030, there will be an estimated 75 million people worldwide living with dementia and this is set to rise to 132 million in 2050.^1,2^ The prevalence of dementia in the Netherlands will increase from 270 000 people with dementia in 2016 to 538 000 in 2040.^3^ Special attention is now needed for the group of first-generation immigrants originally from Turkey, Morocco, and Suriname that constitute the largest immigrant populations in the Netherlands.

The prevalence of dementia among those immigrants will increase twice as fast compared with the native population.^3^ This is the result of demographic changes (the immigrants who came to the Netherlands at the beginning of 1960s have now reached old age) and of specific risk factors for dementia, such as cardiovascular diseases, which have a higher prevalence in this population.^4^ Besides this, the prevalence of dementia is probably higher among these immigrant groups because many of them have received little education, which considerably heightens the relative risk of dementia.^5^ Recent research suggests that dementia might be up to three to four times more prevalent in the majority of non-Western immigrant groups when compared with the native Dutch population.^4 ^GPs, together with other community care services, play a central role in detecting and diagnosing dementia, and organising medical care to patients with mild cognitive impairment or dementia. GPs increasingly have to care for patients with a non-Western immigration background who have dementia. In the Netherlands, the four biggest cities — Amsterdam, Rotterdam, The Hague, and, to a lesser degree, Utrecht — have a large proportion of older non-Western immigrants. Providing tailored primary care is challenging and often hampered by linguistic, cultural, and diagnostic barriers.^6^ Diagnosing dementia is difficult because of the high proportion of low literacy and the low cultural validity of assessment tools.^6–9^ Dementia is still a taboo subject among non-Western immigrants and is often not acknowledged as a disease, but as a side effect of old age.^10^ Care is more often provided by family members than by official organisations compared with native older people.^11^ This often causes a delay in their first presentation to the GP.^10,12^

Specific problems with immigrant populations have only recently been acknowledged as a healthcare priority. There is little research on this topic. Formal guidelines do not give any specific recommendations on this group, or on how the organisation of dementia care services should be developed.^13–15^ More insight into the perspectives of GPs on providing care for immigrant patients with dementia is necessary to improve dementia care. Therefore, the aim of this study was to describe the barriers for providing primary care to immigrant patients (Moroccan, Turkish, and Surinamese) with dementia from the perspectives of GPs. Recommendations for improving primary care for non-Western immigrants with dementia will be reported.

## Method

A sequential explanatory mixed-methods study was conducted. The rationale for this type of mixed-method design was complementarity. The topic that was studied was comprehensive (many barriers in dementia care) and complex (scarce evidence on this issue). It was expected that combining two different research methods (a quantitative survey and a qualitative focus groups study) would result in a comprehensive look at the research problem from many perspectives and could offer a more complete picture.

A quantitative study was chosen to start the research, to asertain which barriers in dementia care for the immigrant patient were reported. The authors wanted to check whether, and to what extent, GPs experienced the same barriers as those described in the available international studies^6–12^ and those reported by experts from specialised memory clinics (as well as elsewhere^6^ by the present authors). With this survey, it was also investigated whether any unknown barriers were reported. Subsequently, focus groups were conducted to further discuss the results from the survey and get more insight on the different barriers.

### Online survey

The questionnaire was constructed by the research team. The theoretical bases was the known barriers described in international studies (summarised in a review written by members of the research team) and the clinical experiences of members of the research team (a specialist in geriatric medicine, a neuropsychologist, and a GP, all of whom worked with immigrant patients).^6^ The questionnaire was discussed several times after repeated input from all members of the research team. When consensus was reached about the format and content of the questionnaire, a colleague was also consulted to reflect on the questionnaire. This was a GP and a member of a local foundation for GPs working in deprived neighbourhoods (members of these foundations were asked to participate in the study). His comments and suggestions for improvement were incorporated into the questionnaire. There was no intention to conduct cognitive pre-tests, because it was not expected that difficulties with the interpretation of the questionnaire or any form of bias would arise. The participants were well educated, the questionnaire was short and easy to fill out (mostly multiple-choice questions), the data were anonymously analysed, and the participants were experts in the field (all working frequently with immigrant patients), so any recall bias or socially desirability bias seemed unlikely. Another important argument was that it was difficult to perform a pre-test in an already very selected population and participants could only be reached by mediation of the contact person from the local foundation.

An online survey was sent to GPs working in deprived urban areas of the four biggest cities of the Netherlands (Amsterdam, Rotterdam, The Hague, and Utrecht). All GPs were members of a local foundation for GPs working in deprived neighbourhoods (that is, 'Achterstandsfondsen') and had a practice population with a high proportion of non-Western immigrants. GPs in Amsterdam, Rotterdam, and The Hague received an email invitation to fill out an online questionnaire from their local professional organisation. GPs from Utrecht were informed about the questionnaire by the monthly newsletter from their professional organisation. The questionnaire was developed in April 2017 using an online programme (www.enquetesmaken.com). The questionnaire contained questions about sociodemographic factors, general practice information, and multiple-choice questions (more than one answer allowed) specifically about experienced barriers in dementia care for immigrant patients. The first invitation was sent in June 2017. A reminder was sent 4 weeks after the initial mailing to participants from Amsterdam, Rotterdam, and The Hague. It was possible to fill out the questionnaire until July 2017. Four gift cards were raffled among participants. Participants were asked if they were interested in joining a focus group to discuss the topic further.

### Focus groups

Subsequently, a focus group study was conducted. Three focus groups were set up in Amsterdam, Rotterdam, and The Hague in September and October 2017. Eight GPs consented to focus group participation in the online survey. Subsequently, recruitment was done using a snowballing technique; that is, each GP that had signed up for participation by the online survey contacted other colleagues who, in turn, nominated colleagues and other contacts.

After analysing the results of the survey, the authors realised that there was a missed opportunity to involve nursing home physicians in the questionnaire, because many GPs mentioned the importance of a good collaboration with these colleagues. In the Netherlands, during the past 10 years, the nursing home physician has acquired an important advisory role for GPs. Therefore, it would also be very interesting to study barriers from nursing home physicians' perspectives, and to discuss their views on collaboration in primary care in the care for the immigrant patient with dementia. One of the participating GPs in The Hague proposed inviting two nursing home physicians with whom she had worked to the focus groups. This was a great opportunity to involve the nursing home physicians in the research project.

The authors aimed to discuss the barriers in dementia care for immigrant patients further and to identify possible solutions and recommendations to improve primary health care.^16,17^ For this purpose, a topic guide was developed to specifically assess these aspects. The results from the survey were integrated in the topic list of the focus groups. The topic guide was also constructed by the research team. Two postdoctoral researchers from the research team developed a draft of the topic list, which was discussed with the research team and adjusted again. Results of the questionnaire were summarised and four main topics and research questions were set for the focus group discussions. Several open questions were formulated with regard to diagnosing dementia, the early detection of dementia, collaboration in dementia care, and the care needs of patients and their caregivers. The topic list was discussed and adjusted until consensus was reached. Next, the topic list was presented to a colleague who specialised in focus group interviewing (a researcher in social science) to make some final adjustments and to further improve the topic list.

The focus group sessions were performed by a trained moderator (GP and postdoctoral researcher), who led the discussion using a fixed protocol, while a researcher observed and took notes (neuropsychologist and postdoctoral researcher).

Anonymised data from the survey were presented to the focus group by theme (diagnosing dementia, the early detection of dementia, collaboration in dementia care, and the care needs of patients and their caregivers) and followed by a statement to initiate the discussion and the reflection on the results of the survey.

The moderator asked questions for clarification, monitored the rules of conduct, and intervened when necessary. To maintain consistency, all focus groups were conducted by the same moderator. After each focus group, both researchers reflected on the session and compared their observations. Each focus group started with a 10-minute introduction, followed by a discussion session of 60 minutes, and a 15-minute wrap-up for the conclusion. The focus groups were audio-taped and transcribed verbatim. From this point, the data were anonymised. The original audio-tapes have been encoded and password protected. The first author and a senior researcher have access to the password. Both the audio-taped data and transcripts will be saved for 15 years to ensure the availability for audits. All participants gave their written informed consent.

### Analysis

#### Quantitative analysis

Statistical analyses of the quantitative data (questionnaires with GPs) were conducted using SPSS (version 23.0). For descriptive statistics, proportions were calculated.

#### Qualitative analysis

The MAXQDA software was used to extract and analyse the data (version 12.0.2). The extracted data were analysed by two members of the research team using thematic analysis.^18^ An open inductive thematic coding approach was performed for data analysis, without the use of a pre-existing coding frame. All transcript passages were coded. After the first focus group, an initial coding frame was created, which was used for the subsequent focus groups. If additional topics emerged, these were added to the coding frame. If the coding differed between the two researchers, consensus was reached after internal discussion. If differences remained, a third researcher was consulted. Axial coding was used for abstracting different codes into more abstract concepts and categories.^17^

## Results

### Baseline characteristics

Of the 480 GPs that were approached to complete an online questionnaire, 76 (16%) agreed to participate. Baseline characteristics are summarised in Table 1. Approximately two-thirds were women (67%), which is higher than the current proportion of female GPs in the Netherlands (48%).^19^ Most of the GPs (74%) monitor only 1–5 immigrant patients with dementia in their current practice.

**Table 1.** Baseline characteristics of GPs (*n* = 76) participating in the online survey

**Working region**	**Amsterdam**	**The Hague**	**Rotterdam**	**Utrecht**	
Frequency, %	37	30	24	9	
**Years of experience**	**0–5 years**	**6–10 years**	**11–15 years**	**>15 years**	
Frequency, %	9	21	22	48	
**Sex**	**Female**	**Male**			
Frequency, %	67	33			
**Reporting high number of patients from given immigrant population**	**Moroccan**	**Turkish**	**Surinamese**	**Other**	
Frequency, %	78	66	51	13	
**Number of immigrant patients for whom dementia care was provided last year**	**0**	**1–2**	**3–5**	**6–10**	**>10**
Frequency, %	18	37	37	7	1
**Number of patients who sought care for dementia-related problems**	**0**	**1–5**	**>5**		
Frequency, %	13	70	17		

The focus groups comprised 17 people in total. Participants were 14 GPs and three nursing home physicians who often worked together with the participating GPs. Nine categories were identified as recurring topics in the focus groups. These were grouped into three major themes: early detection of dementia; the diagnostic barrier; and care needs (Box 1). In the results section, each theme is discussed with data from the survey followed by further elucidation with data from the focus groups. The nursing home physicians contributed in the discussion specifically about collaboration and the diagnosing of dementia.

**Box 1.** Categories and themes

Categories	Themes
1. Early signs and symptoms	1. Early detection of dementia
2. Creating awareness	
3. Dementia as a taboo subject	
4. Diagnostic screening instrument in primary care	2. The diagnostic barrier
5. Role of memory clinics	
6. Other disciplines for diagnosing dementia	
7. Assessment of care needs	3. Care needs
8. Home-care organisations	
9. Informal caregiver stress	

### Barriers to providing care to immigrant patients with dementia

**Figure 1.**
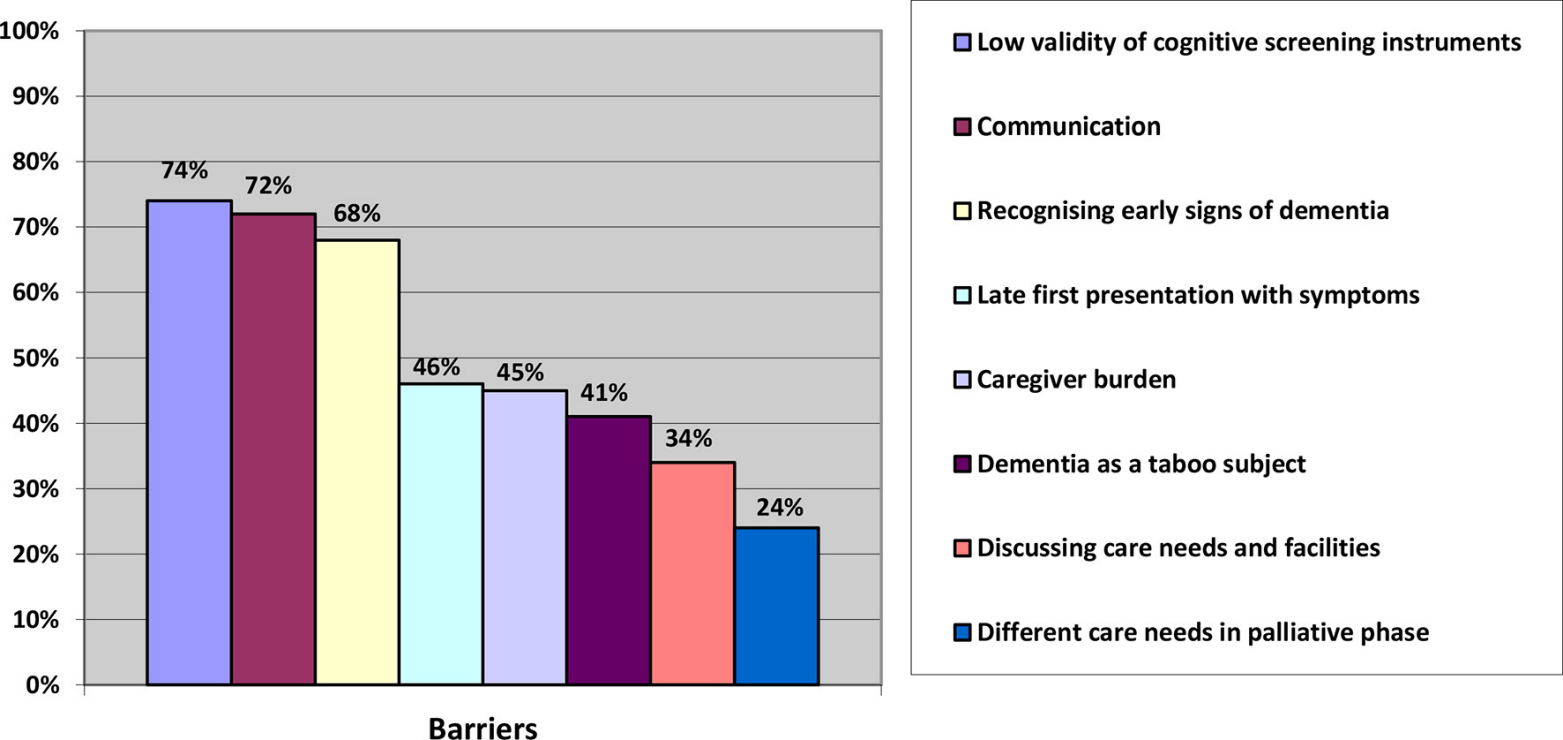
Barriers of providing care to immigrants patients with dementia reported by GPs (*n* = 76)

Results from the survey show that almost 65% of the participants experienced significant barriers in providing care for immigrant patients with dementia and 29% experienced some barriers. Figure 1 summarises the barriers that were reported by the participants (multiple answers were allowed). Most frequently reported were: the low validity of cognitive screening instruments (74%); the language barrier (72%); the difficulty with recognition of early signs and symptoms of dementia with immigrant patients (68%); a late first presentation to the GP (46%); informal caregiver burden (45%); and dementia as a taboo subject (41%).

### Theme 1: early detection of dementia

#### Early signs and symptoms

**Figure 2.**
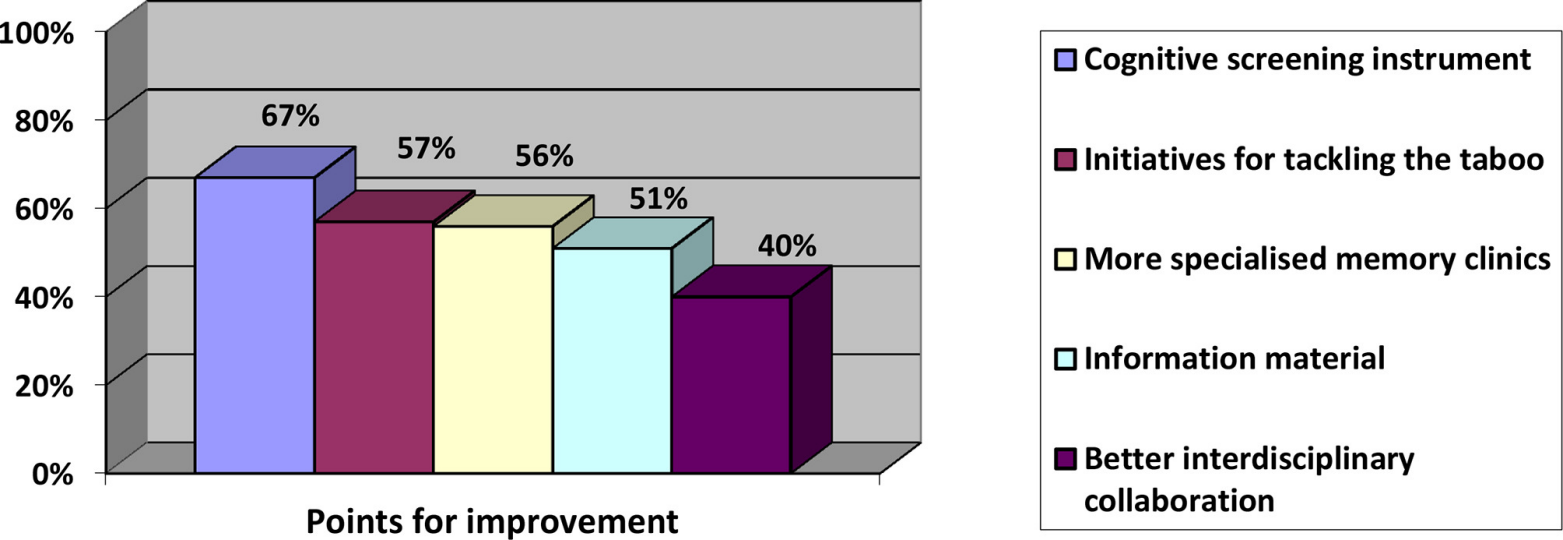
Points for improvement of early signalling of dementia with immigrant patients.

Early signs and symptoms of dementia in immigrant patients that were frequently reported in the online survey were observations or concerns about the wellbeing of the patient expressed by the family caregiver (94%), behavioural changes (67%), memory loss (60%), frequent consultations (54%), increase in unexplained somatic complaints (42%), and other cognitive changes (27%). Almost 31% of GPs reported that early complaints were hardly seen as a result of a late first consultation. According to 84% of the participants, early detection of dementia should be improved. Figure 2 shows reported points for improvement, which will be discussed later.

During the focus group sessions, some GPs shared the opinion that more training and education for GPs is important and needed on this subject. This was supported by the online questionnaire, wherein 93% of the responders agreed on the need for more education. The most important educational topics were: diagnostic screening possibilities (78%); early signs and symptoms (70%); culturally sensitive dementia care (68%); epidemiology (18%); and communication skills (16%). For example, one responder commented:

*'I think it would be very nice if GPs could get training and education on this topic, so we can be more proactive. I think we can do better if the subject gains more of our interest and I think that our work for immigrant patients with dementia will be more satisfying if we are providing better care.'* (FG1, SP4)

#### Creating awareness

The majority of the focus group participants found that GPs play an important role in creating awareness on dementia and in gaining patients’ trust on this subject. Figure 2 shows that 51% of the responders of the survey found that more information and educational material in different languages should be available. During the focus group sessions, the participants were unanimous in their view that — owing to frequent reorganisations, budgetary savings, change in indication criteria and financial compensation — an overview of the existing organisations that provide culturally sensitive care and contact details was missing. The majority of GPs reported a clear need for culturally sensitive communication tools on this subject; only a few of the participants already made use of existing communication tools. During the focus groups, consensus existed on the shared responsibility with other stakeholders, such as municipality and home-care organisations and welfare facilities, for creating more awareness with this specific patient group. Public campaigns, community approaches, and other means of information provision (for example, with Alzheimer 'memory' cafes or information provision by cultural advisers) can be organised by such parties. This was supported by the results of the online survey whereby 40% of the participants found that better interdisciplinary collaboration is needed (Figure 2).

#### Dementia as a taboo subject

Figure 2 shows that 57% reported a high need for initiatives for tackling dementia as a taboo subject among immigrants. In the focus groups, it was discussed that the taboo subject, in combination with an often closed community, makes it difficult to get an opening to talk about dementia. Family caregivers tend to take over many household responsibilities and personal care, even if the patient is still able to do parts of the tasks. They attempt to maintain the facade that the patient is functioning normally in daily life, which hampers the signalling of early signs of dementia for the professional. Four GPs mentioned that financial abuse (the misuse of a personal budget for caregivers) was a reason to postpone dementia diagnostics.

Five GPs brought up the ‘right not to know’ and the discussion of whether early detection of dementia actually results in a health gain for these patients. Many families choose not to inform the patient about the diagnosis, which is not in line with the Western dominant norms regarding communication and autonomy:

*'I wonder, if we are more proactive with this specific group of patients, which of course would be a good thing, if this group will actually benefit. Will there be a health gain if we diagnose dementia more often in immigrant patients? I wonder if there might be a benefit of being kept in ignorance of the diagnosis.'* (FG3, SP2)

### Theme 2: the diagnostic barrier

#### Diagnostic screening instrument in primary care

Figure 2 shows that there was a high need for a validated cognitive screening instrument in the primary care setting (67%). Participants of the focus groups agreed on this. Such an instrument could support GPs with their diagnostic and referral process, as it is often difficult to interpret the reported symptoms from immigrant patients and put them into context. Many participants regarded a screening instrument as a very helpful tool to start the conversation on a taboo subject. For example, one participant stated:

*'Together we can offer a much better diagnostic work-up at home and see what goes wrong in the home situation, than at a specialised memory clinic. At home you observe so much more and this is so valuable. And as a GP or practice-based nurse specialist, you have much easier access and for the patient it’s less threatening. The patient and their family often give back that they find this a very welcome way for a diagnostic work-up. A culturally sensitive diagnostic cognitive screening instrument would then be a good tool to make things more visible and clear.'* (FG2, SP4)

#### Role of memory clinics

Despite the need for a cognitive screening instrument in primary care, the majority (56%) of GPs also felt that more specialised memory clinics are needed (Figure 2). Two important arguments for referral to a specialised memory clinic were raised during the focus groups. The first one was to confirm or rule out the diagnosis, especially in complex patients with other psychiatric symptoms, or with suspected mixed dementia. The second was to meet the patient’s or caregiver's demand for referral. The majority of the participants experienced that immigrant patients prefer further diagnostic work-up by an expert in a secondary care setting with access to diagnostic imaging and treatment options. In this way, the referral was used as a part of the counselling and acceptance process for the patient and their caregiver to subsequently discuss further care possibilities. In contrast, five participants mentioned that some patients prefer diagnostic work-up at home, because patients want to avoid memory clinics given the existing taboo, or they consider the full work-up too heavy a burden. Five GPs mentioned that the involvement of individualised care was not addressed by memory clinics. Two GPs had positive experience with memory clinics that did provide care facilities such as referral to day care, a dementia case manager, and/or home-care:

*'Sometimes you need a specialised memory clinic, especially when it concerns patients with an history of a stroke or who are depressed, because it involves complex presentations, then the specialised memory clinic can support you *[when you consider] *“Is it a passive elderly patient or is it a depressive patient, or does it concern a patient with memory loss? And what is the best way to care for this patient ?".'*(FG1, SP5)

#### Other disciplines for diagnosing dementia with immigrant patients

About half of the participants of the focus groups mentioned that referral to other disciplines, mainly nursing home physicians, geriatric mental health organisations, or occupational therapists could be of added value. These professionals often have an outreaching approach, and assessment of care needs at home is a routine part of their practice. All agreed on the importance of one permanent contact person with the role of care coordinator. This person knows the available healthcare services and is in close contact with other parties. Participants found that a practice-based nurse specialist can play an important role, among other things, by offering low-threshold continuity of care. A periodic dialogue structure might be of benefit. For example, one participant stated:

*'I think, in any case, it is welcome to have a fixed set of people to call or to consult, this is something I miss in my practice at the moment. You have the feeling you’re on your own in solving sometimes very complicated problems.'* (FG2, SP5)

### Theme 3: care needs

#### Assessment of care needs

Care needs of immigrant patients or their caregivers that were most often reported in the survey were information on medical devices and/or supplies (95%); financial compensation for caregivers (61%); information about the course of the disease (49%); diagnosis (45%); deploying home-care facilities (40%); and information about prognosis (36%). Eleven participants from the focus groups reported that finding the specific care needs of immigrant patients with dementia is extremely difficult:

*'To be honest, I never had an immigrant patient with dementia with a specific request for help.'* (FG2, SP2)

They highlight the linguistic barrier and the fact that care is often taken over by family members, who do not often consult the GP for help. GPs, therefore, do not have a good impression of the actual patient’s condition and situation at home. Often, the GP is consulted for the first time when the caregivers are faced with a crisis situation. Most of the GPs argue that a periodic geriatric assessment and an identification of the patient’s condition and care needs is desirable. This creates a certain level of trust and might stimulate discussion of care needs. Half of the participants found that general practice-based nurse specialists or dementia case managers (from home-care organisations) should have an important role within this matter.

**Figure 3.**
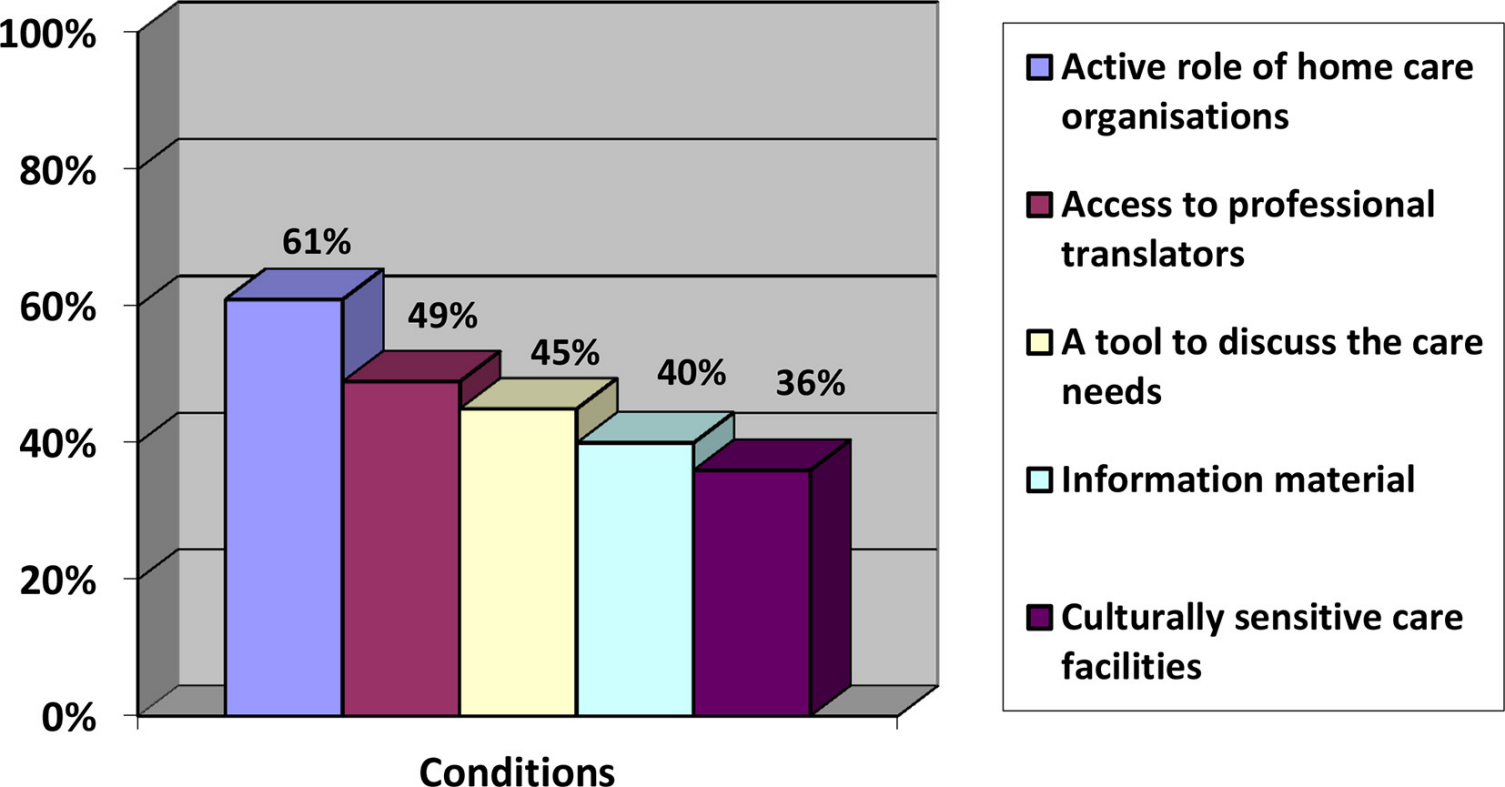
Conditions needed to connect with the care needs of immigrants patients with dementia and their caregiver.

GPs from the online survey formulated different conditions that were needed to connect with the patient and their caregiver care needs (Figure 3). Most frequently mentioned were an active role of home-care organisations (61%), an easy access to professional translators (49%) and a tool to discuss the care needs with the patient and their informal caregiver (45%). The abolishment of financial compensation for professional translators was also reported by the majority of the focus groups participants:

*'In my practice I have many low-literate patients. Fortunately, I have many employees who together speak an unbelievable number of languages. So ad hoc translation is sometimes possible, but I often use a professional translator. Because I am a member of my local foundation for general practitioners working in deprived neighbourhoods, these cost will be compensated. I want to campaign that this will be compensated again for all general practitioners. It’s unacceptable this has not happened yet and can be harmful to patients.'* (GF3, SP1)

#### Home-care organisations

Participants of the focus groups indicated that home-care nurses are poorly accessible for consultation. Most GPs only occasionally receive a report from a nurse or the dementia case manager. Dementia case managers are not always available and seem to lack time to really get to know the patients. One participant had a very positive experience with a Moroccan home-care organisation. Ten of the participants strongly criticised the Turkish and Moroccan home-care organisations as not being reliable and putting financial goals above quality of care:

*'I am surprised about the few contact moments with home-care organisations. I mean they are the "ears and eyes", but they rarely call. Only if a patient has a thick leg, but that will be it. I find it shocking actually. It represents the quality of care, which is so fragmented. It is almost impossible to get in contact with everybody. You can’t keep up. That’s very disappointing.'* (FG2, SP6)

#### Informal caregiver stress

A total of 45% of the participants from the online survey mentioned caregiver burden as an important barrier (Figure 1). Most of the GPs from the focus groups considered it their responsibility to recognise and assess the healthcare needs of caregivers, although they acknowledged that caregivers often disregard GP advice on caregiver stress, which, in turn, can result in a crisis situation. A few GPs considered it the caregiver's own responsibility to consult them for help:

*'Well, bringing up the subject of caregiver stress is difficult and is often done too late. Even when they are completely overloaded, they show up with a smile. If you ask how they are doing they respond: "We don’t need any help from strangers, we do everything ourselves for Mama." And then at some point they suddenly can’t take it any more.'* (FG2, SP3)

They expect caregiver stress to be an increasing problem as more second and third-generation immigrants have to balance caregiving with their own family tasks and career, in a society transitioning to a dual-earner family model. Participants agreed on a clear need for more culturally sensitive nursing homes, day care facilities, and home-care organisations. This should be an important point for the political agenda. Also, it was proposed that more initiatives are needed to inform caregivers about those culturally sensitive facilities and resources:

*'If we get more culturally sensitive nursing homes, it will be easier and more accessible to visit the GP and say: "Yes, we can’t take it anymore, can she go to that nursing home?" After all, they are reassured that the nursing home provides good care and they can practice their own language, culture, and religion. It is less of a barrier to leave their house. So I think it is worth the investment for the government if we want to prevent future problems and crisis situations.'* (FG1, SP 7)

## Discussion

### Summary

This study shows that GPs experience many obstacles in the care for the immigrant patient with dementia. Most important obstacles are the detection of symptoms; dementia as a taboo subject; the lack of a cognitive screening instrument; and family caregiver burden. Proposed recommendations include the availability of a cognitive screening instrument; specialised memory clinics; and initiatives to create awareness among immigrant patients and their caregivers. Initiatives aimed at reducing the burden of the informal caregiver are important, as well as availability of culturally sensitive information provision and care facilities. A professional translator should always be freely accessible, and training and education of healthcare professionals is necessary. A strong collaboration should be pursued with primary healthcare workers, and investment is necessary in home and community care organisations, general practice-based nurse specialists, nursing homes, and geriatric care physicians.

### Strengths and limitations

To the authors’ knowledge, this is the first mixed-methods study that assesses primary care physicians' attitudes towards providing care to immigrant patients with dementia and provides practice recommendations for health care. The results should be interpreted with a degree of caution owing to the relatively low participation rate in the online survey and sample size of the focus groups. Reasons for non-participation were not monitored and, therefore, baseline characteristics could not be compared with those of the participants. The most important reason for non-participation was probably the time burden and the amount of other participation requests for research projects. This issue was addressed by all contact persons of the local foundation for GPs working in deprived neighbourhoods. Therefore, it seems unlikely that baseline characteristics of participants will be very different than those of non-participants. Table 1 shows an even distribution of age, sex, and location among the participants.

As recruiting participants proved to be difficult, most often because the amount of other participation requests impinging on GPs scarce time, the authors choose to conduct three focus groups and not to proceed until thematic data saturation was reached. However, no new themes were added after the second focus group. The authors cannot exclude that the survey findings might have introduced bias in the focus group discussions, although the explanatory design contributed significantly in understanding different barriers in greater detail, and in identifying possible solutions and recommendations. The snowballing technique may also have introduced a selection bias for the focus groups; however, the authors aimed to include participants that were very similar: GPs working in the biggest cities in the Netherlands who offered dementia care, with a practice population with a high proportion of immigrant patients. These GPs know the patients well and are familiar with the problem of offering dementia care to this group. The authors wanted to formulate recommendations to improve health care based primarily on the experiences of these professionals. This might limit the generalisability of the findings, but increases the validity of them.

This study focused on the three largest immigrant groups in the Netherlands. The findings, therefore, cannot be directly extrapolated to other immigrant groups and other European countries, but it can be assumed that many of the reported cultural and linguistic barriers also hold for other immigrant groups.^20^ Furthermore, the generalisability of the current findings may be a limitation, given the extent of diversity between international primary care structures.

### Comparison with existing literature

Barriers that were mentioned by primary care physicians in this study showed many similarities with other studies that investigated patient perspectives, such as dementia as a taboo subject, the need for culturally sensitive care facilities, and insufficient monitoring by their GP.^20–22^ In this study, caregiver burden was mentioned as a serious problem. Wezel *et al* focused on the perspectives of caregivers, and showed that family carers derived a great deal of satisfaction from giving care to a relative with dementia and that this fulfilment largely outweighed the burden of care.^11^ This might reflect that healthcare professionals interpret the caregiver overload from their own viewpoint and standards. This issue was also raised during the focus group when discussing the ‘right not to know’. Cultural assumptions and differences about truth-telling has been extensively studied in other studies, mainly those focusing on palliative or cancer care. While autonomy has gradually become a key concept in the doctor–patient relationship, truth-telling is far from being the norm in many countries in the world. Despite the general agreement on the benefits of open communication between physicians and patients, there is still strong resistance against disclosure of certain diagnoses and prognoses in many cultures. This needs careful attention and is an important educational topic,^23^ especially since 84% of the participants in the present agree that early detection of dementia should be improved. This reflects the huge gap between the perspectives of the doctor and the patient.

### Implications for practice

These findings emphasise the importance of improving primary care to immigrants with dementia. Recommendations should be implemented as soon as possible. Currently, the validation of a short diagnostic cognitive screening instrument, the Dutch version of the Rowland Universal Dementia Assessment Scale, is finished and the feasibility will be studied in general practice.^24^ There is a need for strong collaboration between primary care organisations, specialised memory clinics, and governmental organisations to optimise health care, to strengthen the information provision, to enhance the availability of culturally sensitive facilities, and to invest in the training and education of professionals. Only then will primary healthcare professionals be prepared for the rising number of immigrant patients with dementia, and the many more to come.
